# Association between tooth size and interarch relationships in children with operated complete unilateral cleft lip and palate

**DOI:** 10.1186/s40510-015-0079-8

**Published:** 2015-05-30

**Authors:** Patrícia Bittencourt Dutra dos Santos, Daniela Gamba Garib, Guilherme Janson, Vivian Helena Assis

**Affiliations:** Department of Orthodontics, State University of Rio Grande do Norte, Caicó, RN Brazil; Department of Orthodontics, Hospital of Rehabilitation of Craniofacial Anomalies, Bauru Dental School, University of São Paulo, Bauru, São Paulo Brazil; Department of Orthodontics, Bauru Dental School, University of São Paulo, Bauru, São Paulo Brazil; Hospital of Rehabilitation of Craniofacial Anomalies, Bauru Dental School, Bauru, São Paulo Brazil

**Keywords:** Cleft lip, Cleft palate, Tooth size

## Abstract

**Background:**

To evaluate mesiodistal tooth width of patients with UCLP comparing tooth size in different Goslon Yardstick scores and between cleft and noncleft sides.

**Methods:**

The Department of Orthodontics at Bauru Dental School and Hospital of Rehabilitation of Craniofacial Anomalies – University of Sao Paulo. Hundred forty-four pairs of dental casts of patients with UCLP. These dental casts were divided into 3 groups: group I (patients with Goslon rating of 1 and 2), group II (Goslon rating of 3) and group III (Goslon rating of 4 and 5). The control group consisted of 40 pairs of dental casts of noncleft Class I patients at the same age range. Mesiodistal width of maxillary permanent central incisors, lateral incisors and first molars were measured using a digital caliper. Intergroup comparisons were performed using ANOVA followed by Tukey tests. T tests were used to compare tooth size between cleft and noncleft sides (p <0.05).

**Results:**

Differences for tooth size were observed between individuals with different Goslon Yardstik scores. Mesiodistal widths of maxillary central incisors in subjects of Group III were significantly smaller compared to Group I and to the control group. The lateral incisors at the cleft side were smaller than the antimere.

**Conclusions:**

Mesiodistal tooth size was smaller in poor Goslon yardstick scores. Cleft and noncleft sides demonstrated similar maxillary tooth size except for the lateral incisor.

## Background

Cleft patients have considerably more dental anomalies than non-cleft patients [1]. Certain key genetic disturbances have been implicated in both dental anomalies and clefting, suggesting a shared genetic etiology in some cases [2,3]. Among many dental anomalies reported in CLP patients, small tooth size is frequently reported [3-6]. Besides reduced crown size, cleft patients demonstrated simplified crown morphology and malformed teeth [3]. Sabóia et al. [7] hypothesize that tooth size reductions may be part of the oral cleft phenotypic spectrum.

Foster and Lavelle [8] reported that most permanent teeth are smaller in cleft patients compared with non-cleft individuals. On the other hand, the investigation of Peterka and Mullerova [9] revealed no significant dimensional differences between mesiodistal widths of individuals with cleft lip and palate (CLP, isolated cleft palate and complete unilateral cleft lip and palate (UCLP)) and non-cleft subjects, with the exception of central incisors for males and second molars for females which were smaller in individuals with CLP [10]. Sabóia et al. [7] found smaller tooth size for canines, second premolars and first molars in the maxillary arch, and for incisors and second premolars in the mandibular arch. However, these previous studies’ samples included all types of clefts (cleft lip (CL), cleft lip and palate (CLP), and cleft palate (CP)). Considering that CL/CLP and CP have different etiological background, it is important to evaluate tooth size in each type of cleft separately. Additionally, no previous study investigated whether crown size can affect interarch sagittal relationship in patients with UCLP.

Therefore, the aim of this study was to evaluate mesiodistal tooth width of permanent teeth in patients with complete unilateral cleft lip and palate, by comparing tooth size in different GOSLON Yardstick scores and between cleft and non-cleft sides.

## Methods

The sample size for each group was calculated based on an alpha significance level of 0.05 and a beta of 0.2 to achieve 80% of power to detect a mean difference of 0.94 mm in central incisor size with a 0.6 mm of estimated standard deviation [10]. The sample size calculation showed that 7 patients in each group were needed, and to increase the power even more, it was decided to select 32 patients for groups at least.

This project was approved by the Ethical Committee of the Bauru Dental School. Patient records were anonymized and de-identified prior to analysis. A study sample of 104 Brazilian-Caucasian patients with unilateral complete cleft lip and palate rehabilitated in a single center was retrospectively selected at Hospital of Rehabilitation of Craniofacial Anomalies. A control group of 40 dental casts of non-cleft Class I patients was selected from the growth study center at Bauru Dental School, matched by age and sex with the study sample. The inclusion criteria of the study sample were patients with complete unilateral cleft lip and palate filed at the Hospital from 1999 to 2011, without associated syndromes, with adequate dental casts taken between 8 and 10 years of age available, history of lip repair performed in the first year of life, and palate repair within the second year of life. The primary surgeries had been carried out by five different surgeons using the Millard technique for lip repair and the Van Langenbeck technique for palate repair.

The study sample was divided into three groups according to the GOSLON Yardstick index by a single examiner:

- Group I: 35 patients with GOSLON Yardstick index of 1 and 2 (13 females and 22 males);
- Group II: 32 patients with GOSLON Yardstick index of 3 (8 females and 24 males);
- Group III: 37 patients with GOSLON Yardstick index of 4 and 5 (11 females and 26 males).

**Figure 1:**
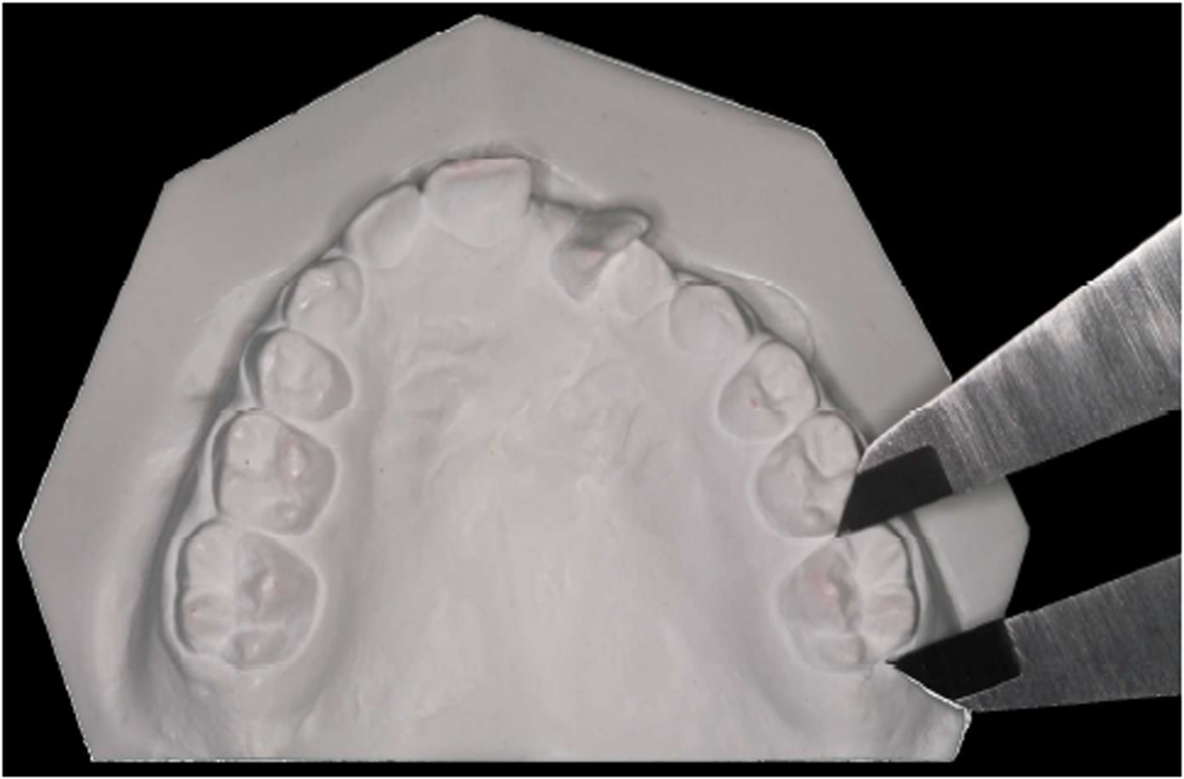
Measurements of crown size in the maxillary arch.

Mesiodistal crown width of maxillary permanent central incisors, lateral incisors, and first molars on both arch sides were measured using a digital caliper (Mitutoyo Corporation, Tokyo, Japan) directly on the dental casts by a single examiner (Figure 1). On the cleft side, only the maxillary lateral incisor distal to the cleft was measured. Teeth with interproximal caries or restorations were not considered.

### Error study

In order to assess the study error, measurements of 30 dental casts were repeated by the same operator, one month after the first evaluation. Random and systematic errors were assessed using Dahlberg’s formula and dependent *t*-tests, respectively.

### Statistical analyses

Normal distribution was evaluated with Kolmogorov-Smirnov tests. Comparisons between male and female and between cleft and non-cleft sides were performed with *t*-tests. Comparison between the experimental groups and the control group was performed with one-way analysis of variance (ANOVA), followed by Tukey tests. Results were considered significant at *P* < 0.05. The statistical tests were performed with SPSS 7.0 for Windows (SPSS Inc., Chicago, IL, USA).

## Results

Results of the error study are shown in Table 1. There were no statistically significant systematic errors and the random errors were within acceptable range.

**Table 1:** **Systematic and casual errors (dependent** ***t*** **-tests and Dahlberg’s formula)**

**Mesiodistal crown diameter**	**First measurement**		**Second measurement**		***P***	**Dahlberg**
	***n*** **= 30**		***n*** **= 30**			
	**Mean**	**SD**	**Mean**	**SD**		
Right central incisor	8.39	0.81	8.46	0.85	0.067	0.13
Left central incisor	8.32	0.01	8.37	0.55	0.090	0.10
Right lateral incisor	6,83	0.68	6.90	0.74	0.469	0.22
Left lateral incisor	6.46	00.1	6.44	0,64	0.667	1.16
Right first molar	10.12	00.1	10.07	0.68	0.540	0.29
Left first molar	9.92	00.1	10.00	0.62	0.240	0.26

In patients with UCLP, there was no difference in tooth size between males and females (Table 2). Crown width of the maxillary central incisors in subjects of Group III was significantly smaller than that of Group I and the control group (Table 3).

**Table 2:** **Intergroup comparison between mesiodistal widths of permanent teeth between the female and male cleft patients (** ***t*** **test)**

**Mesiodistal crown diameter**	**Female cleft**	**Male cleft**	***P***
	**Mean (SD)**	**Mean (SD)**	
Central incisor	8.39 (0.75)	8.54 (0.65)	0.089
Lateral incisor	6.52 (0.71)	6.57 (0.65)	0.615
Molar	9.97 (0.56)	10.06 (0.62)	0.246

**Table 3:** **Intergroup comparison of maxillary tooth size between cleft and non-cleft groups (ANOVA followed by Tukey tests)**

**Mesiodistal crown diameter**	**Group I**	**Group II**	**Group III**	**Control group**	***P***
	**(** ***n*** **= 35)**	**(** ***n*** **= 32)**	**(** ***n*** **= 37)**	**(** ***n*** **= 40)**	
	**Mean (SD)**	**Mean (SD)**	**Mean (SD)**	**Mean (SD)**	
Central incisor	8.63 (0.77)A	8.39 (0.62)AB	8.27 (0.72)B	8.60 (0.60)A	0.004*
Lateral incisor	6.64 (0.75)	6.44 (0.87)	6.31 (0.80)	6.61 (0.52)	0.190
Molars	9.99 (0.54)	10.10 (0.62)	9.95 (0.61)	10.07 (0.63)	0.486

*Statistically significant at *P* < 0.05. Different letters represent statistically significant differences.

The maxillary lateral incisor was smaller on the cleft side (Table 4).

**Table 4:** **Intergroup comparison between mesiodistal widths of permanent teeth between the cleft and non-cleft sides in the UCLP patients (ANOVA followed by Tukey tests)**

**Mesiodistal crown diameter**	**Cleft side**	**Non-cleft side**	**Control group**	***P***
	**Mean (SD)**	**Mean (SD)**	**Mean (SD)**	
Central incisor	8.36 (0.66)	8.45 (0.79)	8.60 (0.60)	0.088
Lateral incisor	5.68 (0.51)A	6.84 (0.65)B	6.61 (0.52)B	0.000*
Molar	10.04 (0.61)	9.99 (0.58)	10.07 (0.63)	0.676

*Statistically significant at *P* < 0.05. Different letters represent statistically significant differences.

## Discussion

It is recognized that poor growth of the maxillary region is related to the effects of primary repair surgery [11], and this is of particular concern for the orthodontist who must correct dentofacial discrepancies during early adolescence. Although those patients who have displayed favorable facial growth may require only relatively routine orthodontic treatment, patients with unfavorable facial growth often need orthognathic surgery for complete correction of dentofacial discrepancies [11]. In our study, all primary plastic surgeries were performed using the same surgical protocol regarding techniques, timing, and sequence. It is important once anteroposterior relationship can be influenced not only by surgery but by surgeon too [12,13].

The GOSLON Yardstick has been shown to be a robust measurement tool with a high degree of reliability and reproducibility and has proven useful for longitudinal assessment of dental arch relationship [14]. Application of the Yardstick is simple and fast, requiring no specialized or expensive equipment. It does not involve application of precise and detailed criteria but relies on a simple method of judgment. The simplicity is its inherent strength, and its longitudinal robustness makes it a valuable tool. The Yardstick considers clinically important variables in all three planes of space and allows ranking of dental casts in order of difficulty to achieve a favorable outcome [15-17]. For this reason, GOSLON Yardstick was used in this study.

No significant intersex difference was found for tooth size in patients with UCLP (Table 2). Previous reports have shown no differences for tooth dimensions between males and females in patients without oral clefts [10,18-20]. Conversely, some authors reported larger teeth in males [7,21-23] as well as in females with oral clefts [8]. Saboia et al. [7] reported that combined mesiodistal widths were consistently smaller in females with oral cleft when compared to males. However, this last study included all types of clefts (CL, CLP, and CP). Rawashdeh et al. [23] found larger teeth in males with UCLP when compared to females, except for maxillary second premolar and mandibular first premolar on the cleft side.

To our knowledge, this study is the first attempt to evaluate mesiodistal tooth size in patients with UCLP with different GOSLON Yardstick scores. The hypothesis is that tooth size could be associated with interarch sagittal relationship. Differences were found for tooth size between patients with different GOSLON Yardistick scores (Table 3). This confirms that the maxillomandibular anteroposterior discrepancy is not the only factor related to GOSLON Yardstick scores. Mesiodistal sizes of maxillary teeth were different in extreme GOSLON Yardstick scores.

The findings indicated that the maxillary central incisors were smaller in Group III as compared to Group I and to the control group (Table 3). The difference indicated a tendency for smaller tooth size in patients with UCLP compared to non-cleft patients and patients with a mild GOSLON score. Foster and Lavelle [8] found that the maxillary teeth of complete UCLP patients were significantly smaller compared to non-cleft controls. On the other hand, Peterka and Mullerova [9] found no differences between mesiodistal tooth widths of individuals with UCLP and a control group. Another study including all cleft types (CL, CLP, and CP) found that maxillary canines and first molars were significantly reduced in size in CLP and CP groups compared to non-cleft subjects [7]. It seems that despite some controversy in this issue, the tendency is for cleft patients to present smaller mesiodistal tooth sizes.

When cleft and non-cleft sides were compared, no differences were observed for permanent central incisors and maxillary first molars. Nevertheless, the maxillary lateral incisor mesiodistal dimension was 1.2 mm smaller on the cleft side compared to the non-cleft side (Table 4). This result is comparable to the published data of Rawashdeh and Bakir [23] showing that the maxillary lateral incisor was statistically significantly smaller on the cleft side. Sofaer [24] and Werner and Harris [3] also demonstrated statistically significant levels of asymmetry occurring between the cleft and non-cleft sides for mesiodistal size of maxillary lateral and central incisors, respectively. According to Sofaer [24], this generalized developmental instability can, to some extent, be genetically controlled, because patients with positive family histories showed some signs of greater asymmetry than those with negative family histories.

Boehn [25] found significant antimere differences in the size of the maxillary permanent incisors in patients with cleft lip and palate [23], suggesting that cleft had a local influence on tooth size. The reason for smaller maxillary lateral incisor on the cleft side in complete UCLP is probably its double embryonic origin [25-28]. Lateral incisor located distal to the alveolar cleft is in fact ‘half of a tooth’ [25-28].

Because patients with poor GOSLON Yardstick scores seem to have reduced tooth size, the orthodontist may leave spaces for composite rehabilitation, to improve sagittal relationship correction. Individual tooth dimensions are important in the clinical assessment of proportions and ratios. Orthodontists aim for an esthetically pleasing dental and facial appearance with a good functional occlusion. To reach these clinical goals, the dentition has to be in proportion; this is important not only from an esthetic standpoint but also occlusally [29].

## Conclusions

Maxillary mesiodistal tooth size was inversely associated with GOSLON Yardstick scores. Similar tooth sizes were found for maxillary teeth at the cleft and non-cleft sides with the exception for the maxillary lateral incisor that was smaller on the cleft side.
